# PCR-based methods for the detection of L1014 *kdr *mutation in *Anopheles culicifacies sensu lato*

**DOI:** 10.1186/1475-2875-8-154

**Published:** 2009-07-14

**Authors:** Om P Singh, Prerna Bali, Janet Hemingway, Sarala K Subbarao, Aditya P Dash, Tridibes Adak

**Affiliations:** 1 National Institute of Malaria Research, Sector 8, Dwarka, Delhi-110077, India; 2 Liverpool School of Tropical Medicine, Pembroke Place, Liverpool L3 5QA, UK; 3 13/704, Eastend Apartment, Mayur Vihar, Phase-I Extension, Delhi-110096, India; 4 World Health Organization, Regional Office for South-East Asia, World Health House, Indraprastha Estate, Mahatma Gandhi Marg, New Delhi-110 002, India

## Abstract

**Background:**

*Anopheles culicifacies s.l*., a major malaria vector in India, has developed widespread resistance to DDT and is becoming resistant to pyrethroids–the only insecticide class recommended for the impregnation of bed nets. Knock-down resistance due to a point mutation in the voltage gated sodium channel at L1014 residue (*kdr*) is a common mechanism of resistance to DDT and pyrethroids. The selection of this resistance may pose a serious threat to the success of the pyrethroid-impregnated bed net programme. This study reports the presence of *kdr *mutation (L1014F) in a field population of *An. culicifacies s.l*. and three new PCR-based methods for *kdr *genotyping.

**Methods:**

The IIS4-IIS5 linker to IIS6 segments of the para type voltage gated sodium channel gene of DDT and pyrethroid resistant *An. culicifacies s.l*. population from the Surat district of India was sequenced. This revealed the presence of an A-to-T substitution at position 1014 leading to a leucine-phenylalanine mutation (L1014F) in a few individuals. Three molecular methods viz. Allele Specific PCR (AS-PCR), an Amplification Refractory Mutation System (ARMS) and Primer Introduced Restriction Analysis-PCR (PIRA-PCR) were developed and tested for *kdr *genotyping. The specificity of the three assays was validated following DNA sequencing of the samples genotyped.

**Results:**

The genotyping of this *An. culicifacies s.l*. population by the three PCR based assays provided consistent result and were in agreement with DNA sequencing result. A low frequency of the *kdr *allele mostly in heterozygous condition was observed in the resistant population. Frequencies of the different genotypes were in Hardy-Weinberg equilibrium.

**Conclusion:**

The Leu-Phe mutation, which generates the *kdr *phenotype in many insects, was detected in a pyrethroid and DDT resistant *An. culicifacies s.l*. population. Three PCR-based methods were developed for *kdr *genotyping. All the three assays were specific. The ARMS method was refractory to non-specific amplification in non-stringent amplification conditions. The PIRA-PCR assay is able to detect both the codons for the phenylalanine mutation at *kdr *locus, i.e., TTT and TTC, in a single assay, although the latter codon was not found in the population genotyped.

## Background

*Anopheles culicifacies s.l*. is the main malaria vector in the Indian subcontinent, affecting mainly rural areas, and contributes 60–65% of malaria cases in India [1]. As this is an endophilic vector, indoor residual spraying (IRS) of DDT is the main strategy used for its control. This species is resistant to DDT in most parts of India. Although DDT is banned in many countries, the recent endorsement by World Health Organization for the use of DDT for IRS for malaria vector control [2] has renewed interest in this insecticide. Pyrethroids are the most commonly used insecticides for IRS and the only insecticide class recommended for impregnation of bed nets due to their relatively low mammalian toxicity and rapid knock down effect on insects. In India, the use of pyrethroids was initiated in 1990s to control malaria epidemics in areas where *An. culicifacies s.l *was resistant to DDT and malathion. Pyrethroid resistance in *An. culicifacies s.l*. was detected in Surat district of Gujarat state, western India, soon after its introduction in vector control programme [3].

DDT and pyrethroids are neurotoxins that act on the voltage-gated sodium channels by modifying their gating kinetics, resulting in the prolonged opening of individual channels leading to paralysis and death of the insect. One of the mechanisms of pyrethroid resistance in insects is referred to as knock-down resistance (*kdr*) caused by reduced target site sensitivity. The phenotype is commonly conferred by a single point mutation (L1014F/S/H) in the IIS6 segment of voltage gated sodium channel [4,5]. Other mutations in different regions of the gene also confer knock-down resistance in some insects [4,6], but among anophelines this is the only locus where point mutations have been reported to date conferring resistance. Only two points mutations have been reported in anophelines at this locus–L1014F in *Anopheles gambiae *[7] (West African *kdr*), *Anopheles arabiensis *[8] and *Anopheles stephensi *[9], and L1014S in *An. gambiae *[10] (East African *kdr*).

The Leu-Phe mutation at the *kdr *locus in *An. culicifacies s.l*. was reported by Hoti *et al *[11] using an allele-specific PCR assay, whose external primers Agd1 and Agd2 were based on *An. gambiae *sequences. However, authors failed to amplify the DNA region of interest using the primers Agd1 and Agd2 (see Additional file 1) due to mismatching with template DNA. As the evident cross resistance between DDT and pyrethroids in this resistant strain strongly suggested a *kdr *type phenotype, an attempt was made to confirm the presence of *kdr*-based insecticide resistance in *An. culicifacies s.l*. by DNA sequencing and developing alternative high throughput methods for *kdr *genotyping. The part of the voltage gated sodium channel spanning IIS4-IIS5 linker to IIS6 segments of *An. culicifacies *species A, B and C was first sequenced which contains at least five residues where mutations have been reported in other insects, namely, Met^918 ^in the IIS4-IIS5 linker, Leu^925^, Thr^929 ^and Leu^932 ^in IIS5 and Leu^1014 ^in IIS6 [6]. Based on the sequences obtained, three PCR based assays for *kdr *genotyping were developed, i.e., Allele Specific-PCR (AS-PCR), Amplification Refractory Mutation System (ARMS) and Primer Introduced Restriction Analysis-PCR (PIRA-PCR). These assays were tested on a DDT/pyrethroid resistant *An. culicifacies *population from Surat district and validated through DNA sequencing. The ARMS and PIRA-PCR were developed as alternatives of AS-PCR, which is often alleged to be non-specific [12-14]. Additionally, the PIRA-PCR was developed as a method by which both the codons of Phe (TTT and TTC) at *kdr *locus can be detected by a single assay.

## Methods

### Study area

*Anopheles culicifacies s.l *were collected from the villages of Surat district of Gujarat state (21–22°N latitude and 73–74°E longitude) located within three Primary Health Centres (PHC), Amladam, Bareda and Dadheda, where a significant level of resistance to DDT and pyrethroids (7–13% and 60–69% mortalities recorded in adults against 4% DDT and 0.05% Deltamethrin, respectively, in WHO's standard insecticide susceptibility tests) have been reported [3]. This *An*. *culicifacies s.l*. population is comprised of species B and C [3]. Indoor resting mosquitoes were collected using hand aspirators between 0600 and 0800 AM. The mosquitoes were preserved in isopropanol and DNA from individual mosquitoes was isolated.

### DNA sequencing

**Figure 1:**
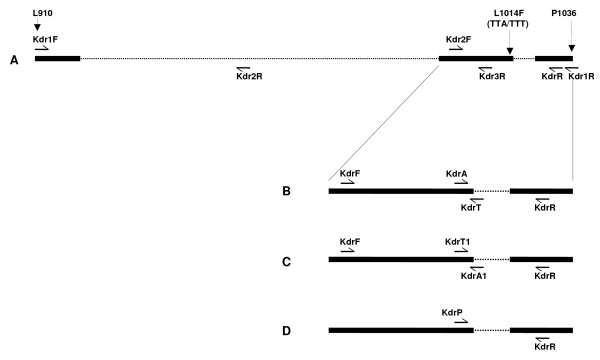
**Schematic diagram showing locations of the primers used for sequencing and PCR based *kdr *genotyping methods**. A: sequencing; B: AS-PCR analysis; C: ARMS analysis; D: PIRA-PCR. Harpoon represents primers; solid and dotted horizontal lines represent exon and intron, respectively.

Initially a 1.45 kb PCR product encompassing IIS4-IIS5 linker to IIS6 segment of voltage gated sodium channel was amplified from individual mosquitoes using primers Kdr1F (5'-CTG AAT TTA CTC ATT TCC ATC A-3') and Kdr1R (5'-TGG TGC AGA CAA GGA TGA AG-3') in a reaction mixture (25 μl) which contained 1× Buffer, 1.5 mM of MgCl_2_, 200 μM of each dNTP, 1.0 μM of each primers and 1.0 unit of AmpliTaq Gold^® ^taq polymerase (Applied Biosystem). The conditions of PCR were: initial denaturation at 95°C for 5 min, followed by 35 cycles of 95°C for 30 S, 48°C for 60 S and 72°C for 120 S, and a final extension step of 72°C for 7 min. The PCR products were re-amplified by semi-nested PCR using primers Kdr1F and KdrR (5'-CGA AAT TGG ACA AAA GCA AAG-3'). The PCR conditions were same as in that of primary PCR. The amplification was checked on 2% agarose gel and PCR product was purified using Millipore's Montage^® ^PCR filter units following vendor's protocol. The purified products were sequenced directly using BigDye Terminator V3.1 (Applied Biosystem). The primers used for sequencing were the same primer used for PCR amplifications, Kdr1F and KdrR. Other internal primers used for sequencing were–Kdr2R (5'-TTG AAA GCC ACG TAC CAT AAC A-3'), Kdr3R (5'-CGG AAC ACA ATC ATG AAG GA-3') and Kdr2F (5'-GAC CAC GAT TTG CCA AGA TG-3') (Figure 1). One specimen of each of *An. culicifacies *species A, B and C were sequenced using these primers. For subsequent sequencing, two PCR fragments were amplified separately using primer sets Kdr1F + Kdr2R and Kdr2F + Kdr1R due to presence of large intron (1 kb) close to the end of IIS5. Both the PCR reactions (25 μl) contained 1× Buffer, 1.5 mM of MgCl_2_, 200 μM of each dNTP, 1.0 μM of each primers and 0.625 unit of AmpliTaq Gold^® ^taq polymerase. The conditions of PCRs were: initial denaturation at 95°C for 5 min, followed by 35 cycles of 95°C for 30 S, 48°C for 45 S and 72°C for 60 S, and a final extension step of 72°C for 7 min. A total of 25 *An. culicifacies s.l*. samples from Surat were amplified using these primers, purified with Qiaquick^® ^PCR purification kit (Qiagen) and sequenced directly using BigDye Terminator. Two specimens were heterozygous for Leu (TTA) and Phe (TTT) at position1014 indicating the presence of a *kdr*-based mechanism in *An. culicifacies s.l*. No other amino acid substitution was detected.

### cDNA sequencing

The total RNA of *An. culicifacies *species A, B and C was isolated using TRIzol Reagent (Invitrogen Corporation) according to the manufacturer's instructions. cDNA synthesis and RT-PCR were carried out using AccessQuick RT-PCR System (Promega Corporation). The first strand cDNA was synthesized by avian myoblastosis virus reverse transcriptase (AMV-RT) at 45°C for 45 min, followed by denaturation of RNA/DNA hybrid and inactivation of AMV-RT at 95°C for 2 min. Second strand cDNA synthesis and subsequent DNA amplification were done by *Tfl *DNA polymerase at the following conditions: 35 cycles of denaturation at 95°C for 30 sec, annealing at 45°C for 30 sec, and extension at 72°C for 1 min followed by a final extension of 5 min at 72°C. The primers used for RT-PCR were Kdr1F and Kdr1R. The product was re-amplified by semi-nested PCR using primers Kdr1F and KdrR, and thereafter sequencing was performed using BigDye Terminator v3.1.

The DNA sequences obtained from cDNA and genomic DNA analyses were deposited in GenBank (accession numbers: GQ268331–GQ268333 and GQ279245–GQ279247).

### PCR based assays

Three molecular methods for *kdr *genotyping were developed based on sequences obtained in this study, viz., Allele Specific PCR (AS-PCR), Amplification Refractory Mutation System (ARMS) and Primer Introduced Restriction Analysis PCR (PIRA-PCR) (Figure 1). In all the PCR-based assays, unless otherwise stated, uniform concentrations of reagents, except primers, were taken, which were: 1× Buffer, 1.5 mM of MgCl_2_, 200 μM of each dNTP and 0.5 unit AmpliTaq Gold^® ^per 15 μl of reaction mixture. All the PCR reactions were carried out in a GeneAmp^® ^PCR System 9700 (Applied Biosystem) thermal cycler. The three assays were tested on field collected *An. culicifacies s.l *for *kdr *genotyping and results were validated following DNA sequencing.

#### Allele-specific-PCR (AS-PCR) analysis

**Figure 2:**
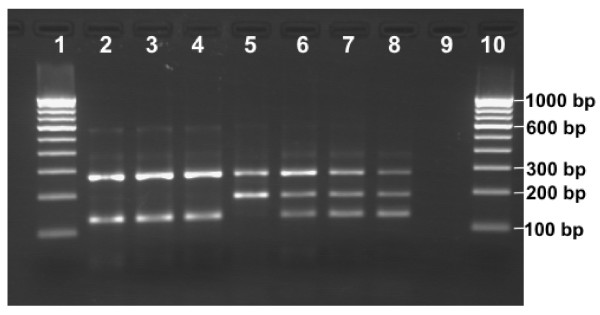
**Allele-Specific PCR (AS-PCR) for *kdr *genotyping**. Lanes 1 & 10: 100 bp DNA ladder; lanes 2–4: SS; lane 5: RR; lanes 6–8: SR; lane 9: negative control

The strategy for designing of AS-PCR was similar to Martinez *et al *[15] and Hoti *et al *[11], but sequences and location of primers were different and were based on *An. culicifacies *sequences obtained through this study. Two external primers flanking *kdr *locus, KdrF (forward, 5'-GGA CCA YGA TTT GCC AAG ATG-3') and KdrR (reverse, 5'-CGA AAT TGG ACA AAA GCA AAG-3'), and two allele specific primers KdrA (forward, 5'-TAC AGT AGT GAT AGG AAA TTT A-3') and KdrT (reverse, 5'-ACT GCT AGG TTA CTT ACG ACA-3') specific for Leu and Phe alleles, respectively, were designed. The predicted sizes of allele-specific bands in PCR product were 127 and 186 bp for Leu (S) and Phe (R) alleles, respectively. A common band of 271 bp was also predicted to be formed by external primers. The scoring of genotypes was made on the basis of presence/absence of 127 and 186 bp bands where presence of 127 bp band only was scored as SS, presence of 186 bp band only as RR, and presence of both the bands as SR. The primer concentrations in PCR reaction were 1.5 μM of KdrF, 0.75 μM of KdrR 3.0 μM of KdrA and 2.0 μM of KdrT. The conditions of PCR were: initial denaturation at 95°C for 5 min, followed by 35 cycles of 95°C for 30 S, 48°C for 45 S and 72°C for 60 S, and a final extension step of 72°C for 7 min. The amplified products were run on 2% agarose gel containing ethidium bromide and visualized under UV in gel documentation system (Figure 2).

#### Amplification refractory mutation system (ARMS)

**Figure 3:**
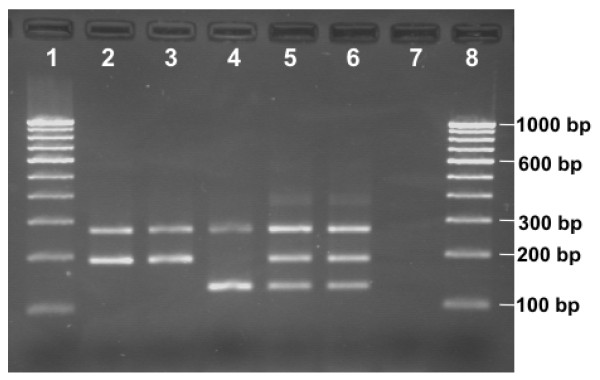
**Amplification Refractory Mutation System (ARMS) for *kdr *genotyping**. Lanes 1 & 8: 100 bp DNA ladder; lanes 2 & 3: SS; lane 4: RR; lanes 5 & 6: SR; lane 7: negative control

The allele-specific primers in AS-PCR are based on single base primer-template mismatch and are prone to non-specific amplification due to mispriming in non-stringent conditions. Amplification Refractory Mutation System is based on incorporation of additional single base mismatch in allele-specific primers near 3' of primer which provides tremendous specificity to SNP (single nucleotide polymorphism) primers [16]. For ARMS the external primers, KdrF and KdrR, were same as in AS-PCR and two allele specific primers, KdrA1 (reverse, 5'-GGG TTA CTG CTA GGT TAC TTA CGg CT -3') and KdrT1 (forward, 5'-CTG GCT ACA GTA GTG ATA GGA AAT cTT-3') specific to Leu (S) and Phe (R) alleles, respectively, were designed using software available at [17] with incorporation of single base mismatch at third base position from 3' end terminus (deliberate mismatched bases in primers are indicated in small letters). The predicted size of a common band, the S-specific and the R-specific bands were 271, 191 and 132 bp respectively. The primer concentrations in the PCR reaction were 1.5 μM of KdrF, 0.6 μM KdrR, 0.6 μM of KdrT1 and 3.0 μM of KdrA1. The conditions of PCR were: initial denaturation at 95°C for 5 min, followed by 35 cycles each of 95°C for 30 S, 48°C for 45 S and 72°C for 60 S, and a final extension step of 72°C for 7 min. The amplified product was run on 2% agarose gel containing ethidium bromide and visualized under UV (Figure 3).

To check the specificity of ARMS, the assay was also performed with increased concentration of MgCl_2 _(up to 3.0 mM) and AmpliTaq^® ^Taq DNA Polymerase (up to 2.5 units per 15 μl reaction mixtures) at an annealing temperature of 45°C.

#### Primer-introduced restriction analysis-PCR (PIRA-PCR)

PIRA-PCR is based on incorporation of a deliberate mismatch in one primer so that a new restriction enzyme recognition site is created in the PCR amplicon in the presence of a specific allele in the target DNA. A PIRA primer KdrP (forward) with sequence 5'-TCC TGG CTA CAG TAG TGA TAG GAA AaT T-3' was designed, where a T-to-A mismatch was incorporated at the 3^rd ^base position from 3' end terminus (shown in small letter in primer sequence). The terminal AAaTT-3' sequence of the PIRA primer (KdrP) creates a recognition site for the restriction enzyme *Apo*I or *Xap*I, i.e., AAATTY, in the amplified PCR product with the primer KdrR (reverse) in the presence of the Phe codon (TTT or TTC) in the template DNA at the *kdr *locus. Hence the PIRA-PCR is capable of detecting both possible codons of phenylalanine (TTT or TTC). With the Leu (TTA) allele, no *Apo*I restriction site is created.

**Figure 4:**
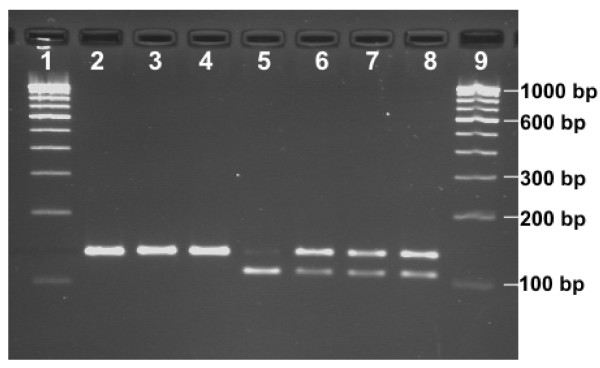
**Primer Introduced Restriction Analysis PCR (PIRA-PCR) for *kdr *genotyping**. Lanes 1 & 9: 100 bp DNA ladder; lanes 2–4: SS; lane 5: RR; lanes 6–8: SR

For PIRA-PCR analysis, a PCR product was amplified using primers KdrP and KdrR with final concentration of 1.0 and 0.5 μM, respectively. The conditions of PCR were: initial denaturation at 95°C for 5 min, followed by 35 cycles of 95°C for 30 S, 48°C for 30 S and 72°C for 45 S, and a final extension step of 72°C for 7 min. Five μL of PCR product was digested with 5 units of *Apo*I restriction enzyme (New England Biolabs Inc) with 1× buffer in a final volume of 20 μl and incubated at 50°C for 1 hour. The restriction products were run on 3.0% agarose gel containing ethidium bromide and visualized under UV illumination (Figure 4).

The predicted size of PCR product amplified with primers KdrP and KdrR is 134 base pairs, which when digested with ApoI or XapI, will be cleaved into 106 and 24 bp fragments (with four bases 5'-overhang) in the presence of Phe allele (TTT/TTC). No cleavage is expected to take place in presence of Leu (TTA) allele. The 134 and 106 bp fragments can be easily resolved on 3% agarose gel whereas 24 bp fragment is normally not expected to be visualized or difficult to discriminate from primer-dimer. Therefore, 134 bp band was scored as S and 106 bp as R.

### Validation of PCR based assays

All the mosquito samples genotyped as RS (n = 18) and RR (n = 1) were sequenced alongside 23 SS in order to validate assays and to check for other mutations in addition to the Leu-Phe change. The primers used for PCR amplification and sequencing were KdrF and KdrR. The sequence data were aligned and analysed using Mega 4 alignment utilities [18].

### Hardy-Weinberg equilibrium analysis

The Exact Test of Hardy-Weinberg equilibrium using a Markov chain on three genotypes, i.e., SS, RS and RR was done using software Arlequin ver 3.11 [19]

## Results

DNA sequencing revealed the presence of a Leu (TTA) to Phe (TTT) mutation at residue L1014 in a total of 19 samples, 18 of which were heterozygote and one homozygous resistant individual. No other mutations were recorded, including L995H mutation reported in Iranian *An. culicifacies s.l*. [20].

Testing of AS-PCR and ARMS assays on *An. culicifacies s.l*. samples from Surat revealed presence of discrete bands of expected sizes, as visualized on agarose gel. With PIRA-PCR also all the expected bands were seen, except 24 bp fragment resulting from the cleavage of PCR product (134 bp). Therefore, the presence of 106 bp cleaved product was scored as R. A total of 186 samples were genotyped using AS-PCR, of which 167 were assigned as homozygous susceptible (SS), one as homozygous resistant (RR) and 18 as heterozygote (SR) based on the criteria mentioned in section 'Methods'. All of the RS and RR, and 80 of the SS samples, as genotyped by AS-PCR, were also assayed with ARMS and PIRA-PCR assays, with similar results to the AS-PCR.

The result of DNA sequencing of 42 samples genotyped by all the three assays (RR = 1, SR = 18, SS = 23) were in agreement with PCR based assays. Thus all three assays are specific and can be used for genotyping.

The AS-PCR, based on a single mismatch at the 3' end of the allele specific primer, was specific and no mispriming was observed. The ARMS was specific even with increased concentrations of MgCl_2 _(3.0 mM) and taq polymerase (3 Units of AmpliTaq Gold per 25 μl reaction).

The observed and expected heterozygote frequencies were 0.09677 and 0.10202 respectively, which did not differ significantly (*p *= 0.41220) from Hardy-Weinberg equilibrium.

## Discussion

Malaria control in India relies on control of the major vector *An. culicifacies*. Knock-down resistance in *An. culicifacies *may be a serious threat to the continued use of pyrethroids, which are currently the insecticide class of choice for control of multiple insecticide resistant vector population by IRS and the only insecticide class available for the impregnation of bed nets. The present study provides the first direct evidence of the presence of the L1014F *kdr *mutation in Indian *An. culicifacies*.

The point mutation at residue L1014 leading to Leu-to-Phe change in voltage gated sodium channel is a most common mechanism associated with knock-down resistance in insects against DDT and pyrethroids. Variant replacements of this residue, L1014H and L1014S, have also been reported [4]. In anophelines, only two mutations, L1014F and L1014S, are reported conferring resistance [7-10]. No mutation at other location has been reported in anophelines except a novel mutation, L995H, reported in a single specimen of *An. culicifacies s.l*. from Iran [20]. During this study, screening of limited numbers of *An. culicifacies s.l*. samples from Surat didn't revealed presence of L995H mutation. The implication of such mutation in DDT/pyrethroid resistance and frequency in resistant population is yet to be investigated.

For monitoring of *kdr *resistance in vector population, there is a need of simple and reliable methods. AS-PCR is a convenient and inexpensive method used for *kdr *detection. This assay format has widely been used with the malaria vector *An. gambiae *in Africa [7,10]. For *An. culicifacies s.l*., an AS-PCR was designed by Hoti *et al *[11] using the external primers Agd1 and Agd2 originally designed for *An. gambiae*. These primers do not work with *An. culicifacies s.l*. from Surat district (see Additional file 1) due to mismatch with template DNA which was revealed after sequencing presented in this study and, therefore, not reliable. Further, a significant departure from HW equilibrium, resulting from deficiency of heterozygotes, had been noticed by the authors, while re-examining genotyping data of *kdr *in *An. culicifacies s.l*. by Hoti *et al *[11], which may be due to discrepancy in genotyping result. This necessitated the development of culicifacies-specific molecular assays for *kdr *genotyping.

The AS-PCR designed for SNP detection is based on single base primer-template mismatch at 3' of the primers. These primers are generally specific but in some instances the mismatched base does not prevent the extension process [12,13]. Mispriming with the primer specific for susceptible (kds) allele has also been reported in *An. gambiae *[21], where the mismatched base is at penultimate position from the 3' terminus. To avoid such mispriming, an ARMS method was developed, where an additional deliberate single nucleotide mismatch is incorporated near the 3' end of the allele-specific primers, which increases the specificity of the ARMS primers [16].

PIRA-PCR is a very reliable method for SNP detection that reduces the possibility of genotyping error due to mispriming. The PIRA-PCR was initially used for genotyping of *kdr *in *An. gambiae *by Janeira *et al *[22], where the mismatch in the PIRA primer was incorporated at the penultimate 3' base in order to create a restriction site. In the PIRA primer designed here for *An. culicifacies s.l*., the mismatch was incorporated at the third base position from 3' end, which seems to be advantageous, being less refractory to amplification in comparison to those where mismatch is at penultimate base. One of the most significant advantages of current PIRA-PCR over previously described methods is its capability to detect both possible codons of phenylalanine, i.e., TTT and TTC, whereas other PCR based assays can detect only one codon. Although in the single mosquito population tested only the A-to-T substitution was encountered, which is the most common substitution in mosquitoes [5], the possibility an A-to-C substitution in other mosquitoes cannot be ruled out and has recently been seen in *Culex quinquefasciatus *where both substitutions (A-to-T/C) exist [23]. It is possible that the A-to-C substitution is being overlooked in other species. The PIRA-PCR strategy described here could be used for screening of Leu-Phe based *kdr *for other mosquito species with little modification in primer sequences. The PIRA-PCR is relatively costly compared to AS-PCR or ARMS, due to involvement of a restriction enzyme step, and it is also slightly more time consuming. The PIRA-PCR may be misleading in case of incomplete digestion of amplified product where RR samples may be scored as RS.

Recently several other molecular methods in addition to PIRA-PCR have been used for *kdr *genotyping as alternative of AS-PCR such as Heated Oligonucleotide Ligation Assays (HOLA) [24], Sequence-Specific Oligonucleotide Probes (SSOP) [25], Fluorescence Resonance Energy Transfer/Melt Curve Analysis (FRET/MCA) [26] and fluorescent based genotyping by capillary electrophoresis [27], but most of these methods are expensive or require more sophisticated instruments. PCR-based methods on the other hand are easy, inexpensive and can be performed in the most of the laboratories equipped with Thermal Cycler.

## Conclusion

The Leu-Phe mutation (L1014F) in the voltage gated sodium channel, which generates the *kdr *phenotype in many insects, was detected in a pyrethroid and DDT resistant *An. culicifacies s.l*. population from Surat, western India. Three PCR-based methods (AS-PCR, ARMS and PIRA-PCR) were developed for *kdr *genotyping based on DNA sequences obtained from *An. culicifacies *species A, B and C. All the three assays were specific. The ARMS method was refractory to non-specific amplification in non-stringent amplification conditions. The PIRA-PCR assay is able to detect both the codons for the phenylalanine mutation at *kdr *locus, i.e., TTT and TTC, in a single assay, although the latter codon was not found in the population genotyped.

## Competing interests

The authors declare that they have no competing interests.

## Authors' contributions

OPS: Conceived strategies for sequencing and PCR-based assays, carried out DNA sequencing and analysis, data analysis and manuscript preparation.

PB: *kdr *genotyping and DNA sequencing.

JH: guided the study and contributed to the manuscript.

SKS, APD and TA: contributed to the manuscript preparation.

All authors provided critical reviews of the manuscript and approved the final version.

## Supplementary Material

Additional file 1 **Evaluation of *An. gambiae*-primers for the amplification of IIS6 segment of the voltage gated sodium channel from *An. culicifacies *(Surat population)** Click here for file
