# Supplementary material for: PCR-based methods for the detection of L1014 kdr mutation in Anopheles culicifacies sensu lato
Source: Malar J. 2009 Jul 14;8:154. doi: 10.1186/1475-2875-8-154 (PMC2717976; doi:10.1186/1475-2875-8-154)
Supplement: Additional file 1 — Evaluation of An. gambiae-primers for the amplification of IIS6 segment of the voltage gated sodium channel from An. culicifacies (Surat population) [file 1475-2875-8-154-S1.doc]

**Figure S1: Evaluation of *An. gambiae*-primers for the amplification of IIS6 segment of the voltage gated sodium channel from *An. culicifacies* (Surat population)**


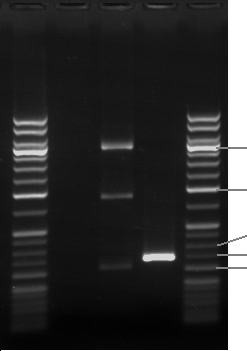


**1.0 kb**

**600 bp**

**320 bp**

**280 bp**

**240 bp**

**1 2 3 4 5**

Lane 1 & 5: DNA ruler

Lane 2 & 3: PCR products amplified with *An. gambiae*-primers (Agd1 and Agd2) using AmpliTaq Gold taq polymerase (lane 2) and taq polymerase from Bangalore Genei (lane 3). It may be noted that the region of interest (predicted size=298 bp) was not amplified with *An. gambiae* primers. However, three spurious amplicons were amplified when normal taq polymerase was used (lane 3).

Lane 4: PCR product amplified with primers designed based on *An. culicifacies* sequence (KdrF and KdrR) using AmpliTaq Gold taq polymerase (predicted size=271 bp).
